# The miR-203a Regulatory Network Affects the Proliferation of Chronic Myeloid Leukemia K562 Cells

**DOI:** 10.3389/fcell.2021.616711

**Published:** 2021-02-15

**Authors:** Jinhua He, Zeping Han, Ziyi An, Yumin Li, Xingyi Xie, Jiabin Zhou, Sihua He, Yubing Lv, Mengling He, Hong Qu, Gexiu Liu, Yuguang Li

**Affiliations:** ^1^Department of Laboratory Medicine, Panyu District Central Hospital, Guangzhou, China; ^2^Department of Hematology, Medical College, Jinan University, Guangzhou, China; ^3^Medical Laboratory of Shenzhen Luohu People’s Hospital, Shenzhen, China; ^4^Department of Hematology, PanYu District Central Hospital, Guangzhou, China

**Keywords:** chronic myeloid leukemia, K562 cells, EGR1, miR-203a, WT1, BMI1, XIAP

## Abstract

To study the molecular mechanism by which miR-203a affects the development of CML, bioinformatics software was used to predict the upstream transcription factors and downstream target genes of miR-203a. A 5’-rapid amplification of cDNA ends assay was performed to detect gene transcription initiation sites. A chromatin immunoprecipitation assay was used to verify the binding of transcription factors and promoter regions. A double luciferase reporter gene vector was constructed to demonstrate the regulatory effect of miR-203a on target genes. Real-time PCR and western blotting were used to detect the relative expression levels of genes and proteins, respectively. The results showed that there was a binding site for the transcription factor EGR1 in the upstream promoter region of miR-203a. WT1, BMI1, and XIAP were identified as target genes regulated by miR-203a. EGR1 and miR-203a were downregulated in human peripheral blood mononuclear cells and the CML K562 cell line, while WT1, BMI1, and XIAP were upregulated. The transcription initiation site of miR-203a was identified in the upstream promoter region (G nucleotide at −339 bp), and the transcription factor EGR1 could bind to the promoter region (at −268 bp) of miR-203a and increase its expression. Over expression of miR-203a inhibited the proliferation of K562 cells. A rescue assay showed that overexpression of WT1, BMI1, and XIAP offset the antitumor effect of miR-203a. Conclusion, EGR1 positively regulated the expression of miR-203a, thus relieving the inhibition of miR-203a on the translation of its target genes (WT1, BMI1, and XIAP) and affecting the proliferation of K562 cells.

## Introduction

Chronic myelogenous leukemia (CML) is a clonal bone-marrow proliferative disease originating from hematopoietic stem cells, which mainly involves granulocytic lineages. CML is characterized by a continuous and progressive increase in the number of peripheral blood leukocytes and the presence of granulocytes in different stages of differentiation, especially neutropenia (Ishida et al., 2019). At present, the traditional chemotherapy drugs for CML are unable to specifically target tumor tissue, which often leads to side effects and adverse reactions, so that chemotherapy is frequently interrupted, resulting in a decrease in the sensitivity of tumor cells to treatment and the formation of early resistance (Iino et al., 2019; Kouhpeikar et al., 2019). Therefore, there is an urgent need to elucidate the molecular mechanism underlying the development of CML.

miRNAs are cell regulatory factors that function at the post-transcriptional level. Under the action of RNA-induced silencing complex, they can combine with specific target mRNAs and play a negative role in regulating gene expression by promoting the degradation of target mRNAs and inhibiting their translation (Kotagama et al., 2015). In CML, increased expression of miR-150 and miR-146a, and reduced expression of miR-142-3p and miR-199b-5p was observed after 2 weeks of TKI treatment, suggesting that this drug has the ability to rearrange the miRNA (Flamant et al., 2010). miR-150 has consistently been observed to be down-regulated across multiple studies making it a promising candidate for early CML diagnosis (Fallah et al., 2015).

Recently, a lack of microRNA (miRNA; miR)-203a expression was found to be closely related to the occurrence and development of CML (Chim et al., 2011; Shibuta et al., 2013). We reported that miR-203a has an obvious anti-leukemia effect and can improve the sensitivity of CML K562 cells to chemotherapy drugs (He et al., 2013). In the present study, bioinformatics software (ChIPBase, PubMed, miRmap, miRanda, miRWalk, PITA, and TargetScan) was used to predict the upstream transcription factors and downstream target genes of miR-203a. The results showed that there were binding sites in the upstream promoter region of miR-203a for the transcription factor early growth response 1 (EGR1), while Wilms tumor 1 (WT1), B cell-specific Moloney murine leukemia virus integration site 1 (BMI1), and X-linked inhibitor of apoptosis protein (XIAP) were identified as potential targets regulated by miR-203a.

In the present study, we aimed to determine whether EGR1 binds to the promoter region of miR-203a and regulates its expression; to confirm whether WT1, BMI1, and XIAP are target genes regulated by miR-203a; and to further clarify that the EGR1-miR-203a-target genes (WT1/BMI1/XIAP) regulatory network participates in the occurrence and development of CML. This study provides a new theoretical basis for elucidating the molecular mechanism of CML and a novel intervention strategy for targeted gene therapy of leukemia.

## Materials and Methods

### Bioinformatics Analysis

Online transcription factor prediction software (ChIPBasev2.0)^1^ was used to identify potential transcription factors that could bind to miR-203a. The PubMed online database^2^ was used to screen the transcription factors related to CML. The common target genes of miR-203a were predicted simultaneously by five miRNA target gene prediction software packages (miRmap, miRanda, miRWalk, PITA, and TargetScan). Finally, PubMed was utilized to screen the target genes with the highest prediction score and assess their relationship with CML.

### Patient Information

Twenty patients with CML admitted to Panyu District Central Hospital, Guangzhou, China from March 2017 to December 2018 were selected as the study subjects (male: 12, female: 8, Age: 53.05 ± 19.7). They were all confirmed to have CML by bone marrow and peripheral blood BCR/ABL gene examinations, and all patients were in the initial stage. Twenty healthy individuals (male:20, Female:0, Age: 32.7 ± 6.3; BCR/ABL(+): 77.42 ± 37.01) from the Health Management Center of Panyu District Central Hospital, Guangzhou, China were selected as controls for the extraction of peripheral blood nucleated cells by using red blood cell lysis. This study was carried out after informed consent was obtained from all subjects and was approved by the Ethics Committee of Panyu District Central Hospital.

### Cell Culture, Transfection, and Cell Grouping

Human CML K562 cells and 293T cells were cultured in Dulbecco’s modified Eagle medium/F12 medium (Invitrogen, Carlsbad, CA) containing 10% newborn bovine serum at 37°C and 5% CO_2_ in an incubator and passaged every 2–3 days. Cells in the logarithmic growth phase and with a trypan blue rejection rate > 95% were used in all experiments. The cells were transfected with small interfering RNA (siRNA) and miRNA mimic sequences according to the instructions of the Lipofectamine 2000 Transfection Kit (Thermo Fisher Scientific, Waltham, MA). The following experimental groups were used: cell blank control group (cells); negative control (NC) group (NC sequence + Lipofectamine 2000); si-NC group (siRNA for NC sequence + Lipofectamine 2000); si-EGR1 group (siRNA sequence for EGR1 + Lipofectamine 2000); miR-203a mimic group (miR-203a mimic + Lipofectamine 2000); si-WT1 group (siRNA sequence for WT1 + Lipofectamine 2000); si-BMI1 group (siRNA sequence for BMI1 + Lipofectamine 2000); and si-XIAP group (siRNA sequence for XIAP + Lipofectamine 2000). The siRNA sequences are shown in Table 1. The sequence for miR-203a was obtained from the website of the National Center for Biotechnology Information^3^.

**TABLE 1:** siRNA sequences.

**Gene**	**siRNA(5’→3’)**
XIAP	1: GACATCAATAAGGAAGAAGAATT
	2: TTCCTAATTGCTTCTTTGTTTTG
	3: GACTTCATTACAGAAAGAGATTA
BMI1	1: CTCCAAGATATTGTATACAAATT
	2: CAGATTGGATCGGAAAGTAAACA
	3: AGGAACCTTTAAAGGATTATTAT
WT1	1: GGCTGCAATAAGAGATATTTTAA
	2:AGGACTCATACAGGTAAAACAAG
	3:GGCCAAGTTGTCAGAAAAAGTTT
EGR1	1:CCGGTTACTACCTCTTATCCATC
	2: CCGGTTACTACCTCTTATCCATC
	3: CGGTTACTACCTCTTATCCATCC
si-NC	GAACTGGGGTGCGTGTGATTT

### Construction of Overexpression Vectors

The following experimental groups were used: NC group (empty vector, pcDNA3.1); pcDNA3.1 + WT1 group (including pcDNA3.1 + WT1 full sequence); pcDNA3.1 + XIAP group (pcDNA3.1 + XIAP full sequence); and pcDNA3.1 + BMI1 group (pcDNA3.1 + BMI1 full sequence). Total RNA was extracted, cDNA obtained by reverse transcription was amplified by polymerase chain reaction (PCR), and the target fragment was recovered using a Gel Recovery Kit (Omega Bio-tek, Norcross, GA). The pcDNA3.1 plasmid and target fragment from gel recovery were ligated using T4 ligase (Qiagen, Hilden, Germany) at 22°C for 3 h. Three single colonies were selected for shake culture, and the positive clones identified by enzyme digestion were sequenced.

### Real-Time (RT)-PCR

RNA was extracted with the TRIzol reagent (Invitrogen), and cDNA generated by reverse transcription with Moloney murine leukemia virus (Invitrogen) was used as the template for PCR (primers are shown in Table 2). miRNA levels were normalized according to the levels of small nuclear U6; mRNA levels were normalized according to GAPDH expression. Relative expression levels were determined by the 2^–Δ^ ^Δ^ ^CT^ method.

**TABLE 2:** Primers for RT-PCR.

**Gene**	**Primer (5’–3’)**	**Product length (bp)**
miR-203a	F:ACACTCCAGCTGGGGTGAAATGTTTAGGACCA	
	R: CTCAACTGGTGTCGTGGA	72
WT1	F: CGATAACCACACAACGCCCA	
	R: GGGCGTTTCTCACTGGTCTC	147
BMI1	F: TGCTGATGCTGCCAATGGCT	
	R: TCTTTGTTTACTTTCCGATC	141
XIAP	F: GGCTTTTATCTTGAAAATAG	
	R:GCATGTGTCTCAGATGGCCT	128
GAPDH	F: GCTCATTTGCAGGGGGGAG	
	R: GTTGGTGGTGCAGGAGGCA	138
U6	F:CTCGCTTCGGCAGCACA	
	R:AACGCTTCACGAATTTGCGT	89
EGR1	F:GGGCCTGGGCACCCCAGACC	
	R:TGAGTGGCAAAGGCCTTAAT	102

### MTT Assay

Cell concentration was adjusted to 1.0 × 10^5^ cells/mL with serum-free Dulbecco’s modified Eagle medium/F12 culture medium; 96-well plates were inoculated with 100 μL/well. After 24, 48, and 72 h of culture, 20 μL MTT solution (Solarbo, Beijing, China) was added to each well. Incubation was continued for 4 h, centrifugation was carried out, and the culture supernatant in each well was removed. Then, 150 μL DMSO was added to each well and the plate was shaken for 10 min to ensure that the formazan crystals were in solution. The optical absorption value of each well was measured at 490 nm.

### BrdU Assay

K562 cells (1–5 × 10^6^) were collected by centrifugation in a 1.5 mL centrifuge tube, and the cells were resuspended gently in 0.5 mL 4% paraformaldehyde fixative and fixed for 10 min. The fixative solution was removed after centrifugation and the cells were washed twice with 1 mL of phosphate-buffered saline (PBS) or 0.9% NaCl for 3 min. After centrifugation, most of the liquid was removed, with about 50 μL remaining. The cells were resuspended gently, applied to a slide and allowed to dry. Then, 100 μL of 50 μM BrdU medium was added to the slide and incubated for 4 h. The slide was washed 3 times with PBS, covered with 5% normal rabbit serum, and nucleic acids were denatured in formamide at 100°C for 5 min. After cooling in an ice bath, the slide was washed with PBS, and nuclei were stained with DAPI in the dark at room temperature for 10 min. The DAPI-stained slides were washed 3 times with PBS for 10 min. The nuclei of BrdU red-positive cells and DAPI blue-stained cells were counted and analyzed. Using Image J software to count, each dot represents a cell, and the results of cumulative counting were analyzed. The experiment was repeated for 3 times, each group randomly selected 10 visual fields to count, according to the results of statistical analysis.

### 5’ Rapid Amplification of cDNA Ends (RACE)

The following primers were used for 5’-RACE: outer primer, 5’-TCGCCGCGCCCGCCGGGTCTA-3’; inner primer, 5’-CTGGACCCAGCGCGCGAGTCC-3’; and connector primer, 5’-GGCCACGCGTCGACTAGTAC-3’. Using a Clontech Smarter RACE cDNA Amplification Kit (Takara Bio, Kusatsu Japan), the upstream primer was adjusted to 2.5 μL and the gene-specific primer was adjusted to 0.5 μL in a total volume of 25 μL. The PCR conditions were 30 cycles of denaturation at 95°C for 30 s, annealing at 68°C for 30 s, and extension at 72°C for 3 min. The PCR products were electrophoresed on a 1% agarose gel, recovered from the gel, and cloned into a T vector for sequencing. The sequencing results and genome sequence were compared to determine the gene transcription starting site (He et al., 2016).

### Chromatin Immunoprecipitation (ChIP)

K562 cells were treated with formaldehyde and incubated for 10 min to generate DNA-protein cross-links. The cell lysates were sonicated to generate chromatin fragments of 200–1,000 bp and immunoprecipitated with EGR1 or IgG as control. The precipitated chromatin DNA was recovered and analyzed by RT-PCR (He et al., 2018). The primers for the promoter region of miR-203a were forward, 5’-CCGCATAAAAGGCGCCGCCG-3’ and reverse, 5’-TCCCAGCCGCCAAGCCCAGC-3’.

### Construction of Double Luciferase Reporter Gene Vectors

The following experimental groups were used: blank group (cells); miR-203a group (miR-203a mimics + psiCHECK-2-WT1-3’ untranslated region [UTR]/psiCHECK-2-BMI1-3’-UTR/psiCHECK-2-XIAP-3’-UTR); miR-203a inhibitor group (miR-203a inhibitor + psiCHECK-2-WT1-3’-UTR/psiCHECK-2-BMI1-3’-UTR/psiCHECK-2-XIAP-3’-UTR); NC group (NC sequence + psiCHECK-2-WT1-3’-UTR/psiCHECK-2-BMI1-3’-UTR/psiCHECK-2-XIAP-3’-UTR); and NC inhibitor group (NC sequence inhibitor + psiCHECK-2-WT1-3’-UTR/psiCHECK-2-BMI1-3’-UTR/psiCHECK-2-XIAP-3’-UTR). The 3’-UTR sequences of the target genes (WT1, BMI1, and XIAP) were amplified using genomic DNA as the template, and *Xho*I and *Not*I digestion sites and protective bases were introduced. The PCR products were subcloned downstream of the luciferin gene of the psiCHECK-2 plasmid. The resulting recombinant vectors were named psiCHECK-2-WT1-3’-UTR, psiCHECK-2-BMI1-3’-UTR, and psiCHECK-2-XIAP-3’-UTR. The recombinant plasmid “seed region” was mutated at a specific site, and the resulting mutated plasmids were named psiCHECK-2-WT1-3’-UTR-mut, psiCHECK-2-BMI1-3’-UTR-mut, and psiCHECK-2-XIAP-3’-UTR-mut. The constructed expression plasmids were identified by double enzyme digestion and sequencing. Luciferase activity was measured using a Dual Luciferase Reporter Assay System Kit (Promega, Madison, WI) on a Tecan M200 luminescence reader according to the manufacturer’s instructions (He et al., 2019).

### Western Blotting

K562 cells of different experimental groups were collected, and protein was extracted using a Nuclear Extraction Kit. Protein concentration was measured by the BCA method. Samples containing 100 μg protein were separated by 10% polyacrylamide sodium dodecyl sulfate gel electrophoresis and transferred to a PVDF membrane. The PVDF membrane was incubated in PBS solution containing 5% skimmed milk powder for 2 h at room temperature and 4°C overnight to block non-specific binding sites. The membrane was incubated with the corresponding antibodies at room temperature for 3 h, washed 3 times with PBS-Tween 20, and incubated at room temperature for 1 h with a horseradish peroxidase-labeled mouse secondary antibody. The protein bands were analyzed by AlphaEaseFC software. quantification has been performed by semi-quantitative. Anti-Bmi1 antibody (abcam: ab182910); Anti-Wilms Tumor Protein antibody (abcam: ab180840); Anti-XIAP antibody (abcam: ab28151); Anti-EGR1 antibody (abcam; ab55160); Anti-GAPDH (AKsomics:KC-5G5).

### Rescue Assay

K562 cells were co-transfected with miR-203a mimic and pcDNA3.1 + WT1, pcDNA3.1 + BMI1, or pcDNA3.1 + XIAP, and K562 cell proliferation was detected by an MTT assay as described in Section “MTT Assay.” K562 cells transfected with miR-203a mimic or miR-203a inhibitor were co-transfected with pcDNA3.1 + EGR1, and the relative gene expression of miR-203a, BMI1, and XIAP was detected by RT-PCR as described in section “Real-Time (RT)-PCR.”

### Statistical Analysis

Data are expressed as the mean ± standard deviation and were processed using SPSS statistical software version 20.0 (SPSS, Chicago, IL). Parametric data can be analyzed by one-way analysis of variance (ANOVA). Non-parametric data should be analyzed by a Kruskal-Wallis test. *p* < 0.05 considered to indicate statistical significance.

## Results

### Bioinformatics Analysis Indicates a Binding Site for EGR1 in the Upstream Promoter Region of miR-203a, and WT1, BMI1, and XIAP Are Potential Target Genes Regulated by miR-203a

**FIGURE 1:**
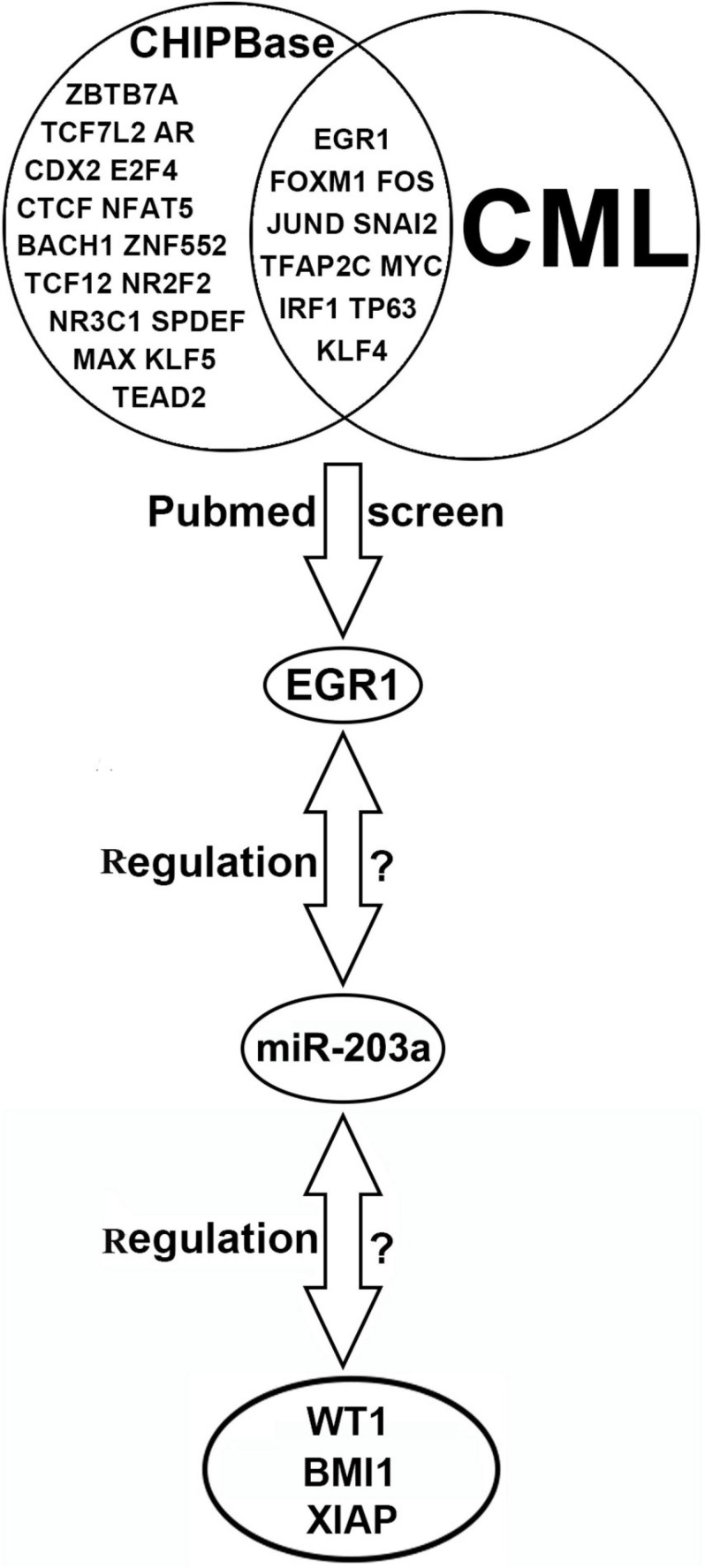
Bioinformatics software prediction of transcription factors binding to miR-203a and miR-203a target genes.

ChIPBase software predicted that 27 transcription factors could bind to the upstream promoter region of miR-203a. By using PubMed, 10 transcription factors related to CML were further screened out, and EGR1, a transcription factor identified in this study, was selected for further analysis. Five common miRNA target gene prediction.

software packages (miRmap, miRanda, miRWalk, PITA, and TargetScan) were used simultaneously to predict 1139 common target genes of miR-203a. Further screening was performed on the 50 target genes with the highest prediction score by assessing their relationship with CML using PubMed. The results showed that Cbp/p300-interacting transactivator 2, tyrosine-protein phosphatase non-receptor type 2, WT1, and 22 other target genes were involved in the occurrence and development of CML. Due to the negative regulatory relationship between miRNAs and their target genes, we screened out the target genes that were expressed at a high level in CML as candidate genes, and the WT1, BMI1, and XIAP genes were finally selected for further study (Figure 1).

### EGR1 and miR-203a Are Downregulated in Human Peripheral Blood Mononuclear Cells and the K562 Cell Line, Whereas WT1, BMI1, and XIAP Are Upregulated

**FIGURE 2:**
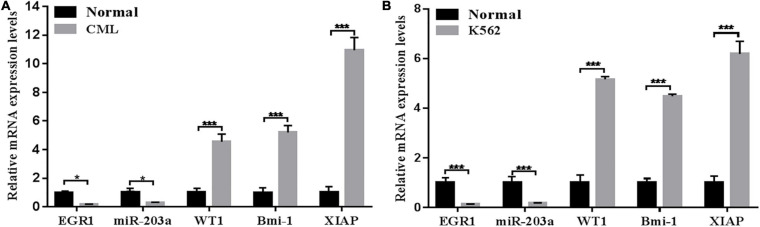
Relative expression levels of genes detected by RT-PCR **(A)** RT-PCR was used to detect the relative expression levels of genes in the peripheral blood of patients with CML. *P < 0.05 and ***P < 0.01 compared with the normal group, *n* = 3. **(B)** RT-PCR was used to detect the relative expression levels of genes in the CML K562 cell line. ***P < 0.05 compared with the normal group, *n* = 3.

The expression levels of EGR1, miR-203a, WT1, BMI1, and XIAP in peripheral blood mononuclear cells and the K562 cell line were detected by RT-PCR. The results showed that the expression levels of EGR1 and miR-203a were downregulated in human peripheral blood mononuclear cells and the K562 cell line, whereas the expression levels of WT1, BMI1, and XIAP were upregulated (Figure 2).

### The Transcription Start Site in the Upstream Promoter Region of miR-203a Is Located at −339 bp, and the EGR1 Binding Site Is Located at −268 bp

**FIGURE 3:**
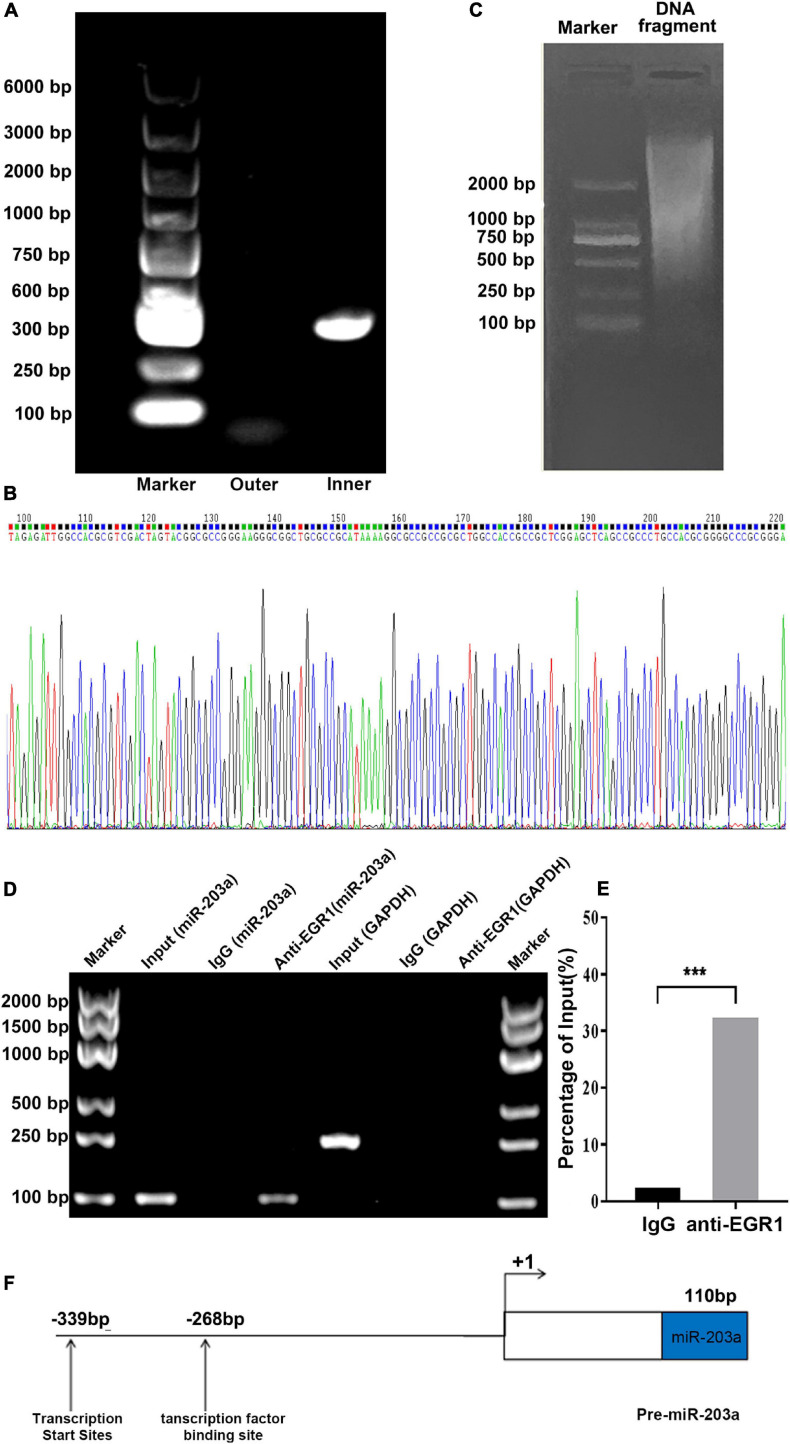
Transcription initiation site for miR-203a determined by 5’-RACE and ChIP assays to assess EGR1 binding to the promoter region of miR-203a **(A)** DNA electrophoresis of 5’-RACE products. **(B)** Sequencing diagram of 5’-RACE products **(C)** DNA electrophoresis of miR-203a. **(D)** ChIP electrophoresis gel. **(E)** Binding of EGR1 to the promoter region of miR-203a, ***P < 0.05, compared with the IgG group, *n* = 3. **(F)** Structure of the miR-203a promoter region with the putative binding site for EGR1.

Specific primers for the miR-203a gene were designed for PCR amplification (Figure 3C). An amplification product of 387 bp was obtained and cloned into the T vector, followed by enzyme digestion. Sequencing indicated that the transcription initiation site of miR-203a was at the G nucleotide located 339 bp upstream of the mature sequence (Figures 3A,B). EGR1 is a nuclear transcription factor that plays an important role in the regulation of cell growth, differentiation, development, and proliferation and in the inflammatory response (Zwang et al., 2012). To confirm that EGR1 can bind to the promoter region of miR-203a, the binding sites predicted by bioinformatics analysis were assessed by ChIP. ChIP electrophoresis showed that there were positive bands in the anti-EGR1 antibody group, but not in the negative control IgG group (Figure 3D). RT-PCR showed that the relative expression of DNA isolated with the anti-EGR1 antibody amplified by the EGR1 ChIP forward/reverse primers was 32 times higher than that of the negative control group, suggesting that the DNA isolated with the anti-EGR1 antibody contained the predicted miR-203a promoter sequence (Figure 3E). These results showed that EGR1 can bind directly to the promoter region of miR-203a. On the basis of these results, we concluded that the transcription start site of the promoter region of miR-203a was located at −339 bp, and the EGR1 binding site was located at −268 bp (Figure 3F).

### miR-203a Negatively Regulates WT1, BMI1, and XIAP Expression by Targeting Their 3’-UTRs

**FIGURE 4:**
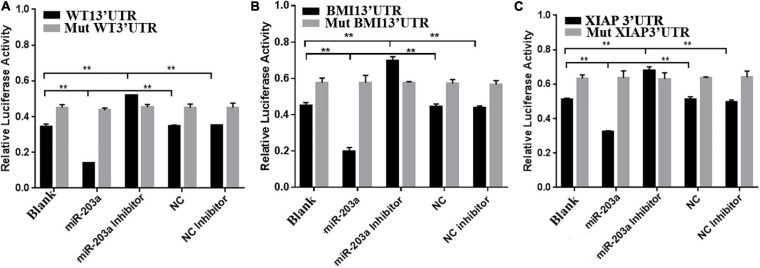
miR-203a binding with the 3’-UTRs of WT1, BMI1, and XIAP **(A)** Comparison of luciferase activity in cells transfected with the 3’-UTR of WT1, **P < 0.05, *n* = 3. **(B)** Comparison of luciferase activity in cells transfected with the 3’-UTR of BMI1, **P < 0.05, *n* = 3. **(C)** Comparison of luciferase activity in cells transfected with the 3’-UTR of XIAP, **P < 0.05, *n* = 3.

**FIGURE 5:**
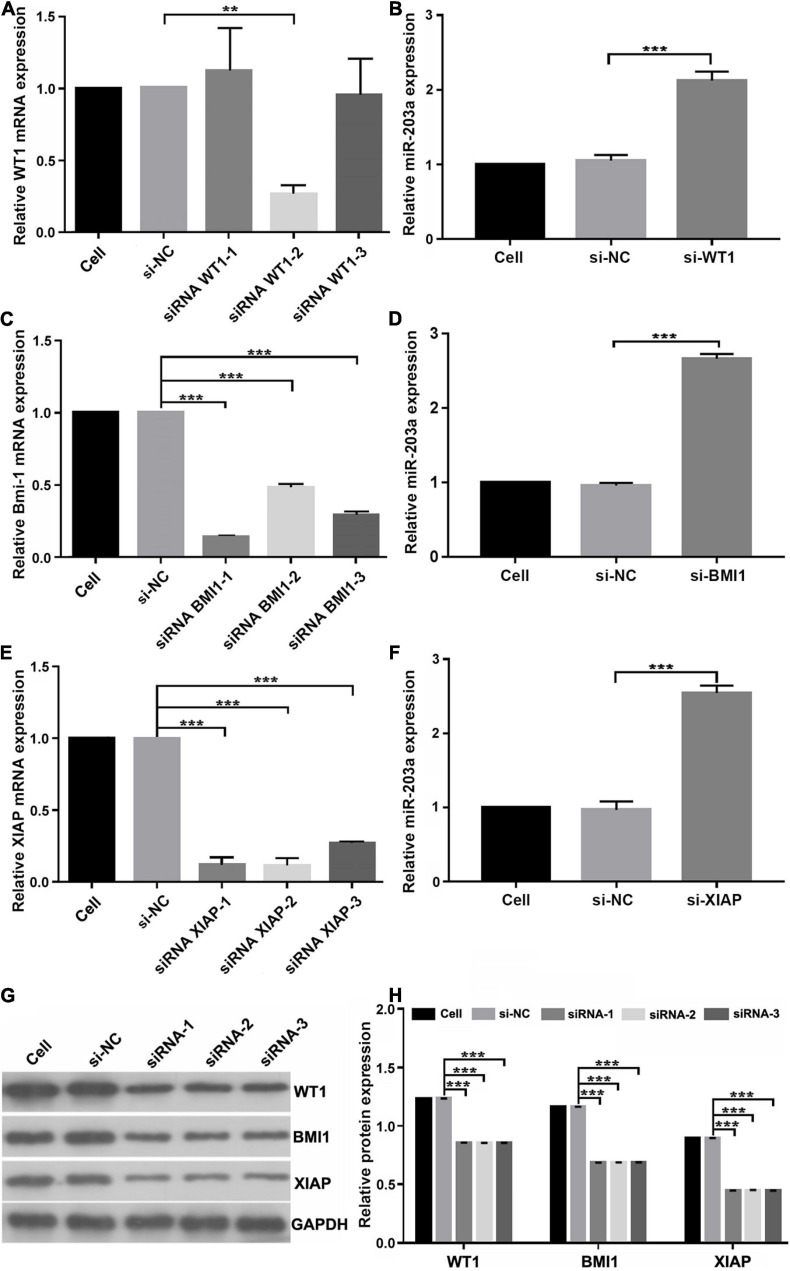
miR-203a negatively regulates WT1, BMI1, and XIAP expression **(A)** Screening of si-WT1 sequences. After transfection of K562 cells with three different siRNAs targeting WT1, si-WT1-2 significantly inhibited the expression of WT1, **P < 0.05 compared with si-NC and si-WT1-1 group, *n* = 3. **(B)** The relative expression of miR-203a was detected by RT-PCR after transfection with si-WT1-2, ***P < 0.05 compared with si-NC group, *n* = 3. **(C)** Screening of si-BMI1 sequences. After transfection of K562 cells with three different siRNAs targeting BMI1, si-BMI1-1 significantly inhibited the relative expression of BMI1, ***P < 0.05 compared with si-NC group, *n* = 3. **(D)** The relative expression of miR-203a was detected by RT-PCR after transfection with si-BMI1-1, ***P < 0.05 compared with si-NC group, *n* = 3. **(E)** Screening of si-XIAP sequences. After transfection of K562 cells with three different siRNAs targeting XIAP, si-XIAP-2 significantly inhibited the relative expression of XIAP, ***P < 0.05 compared with the si-NC group, *n* = 3. **(F)** The relative expression of miR-203a was detected by RT-PCR after transfection with si-XIAP-2, ***P < 0.05 compared with cell and si-NC groups, *n* = 3. **(G)** The relative protein expression of WT1, BMI1, and XIAP was detected by western blotting, *n* = 3. **(H)** Relative protein expression of WT1, BMI1, and XIAP, ***P < 0.05 compared with the si-NC groups, *n* = 3.

To determine whether miR-203a targets the 3’-UTRs of WT1, BMI1, and XIAP, 293T cells were co-transfected with luciferase reporter plasmids containing the wild-type or mutant 3’-UTR of WT1, BMI1, or XIAP and miR-203a mimic. Co-transfection of 293T cells with miR-203a mimic and wild-type 3’-UTR plasmids of WT1, BMI1, and XIAP inhibited luciferase activity (*P* < 0.05). However, co-transfection of 293T cells with miR-203a mimic and the 3’-UTR plasmids of mutant WT1, BMI1, and XIAP had no effect on luciferase activity (*P* > 0.05). These results showed that miR-203a could target the 3’-UTRs of WT1, BMI1, and XIAP and play a regulatory role (Figures 4A–C). After the expression levels of WT1, BMI1, and XIAP were downregulated in K562 cells, the expression levels of miR-203a were found to be upregulated. These results showed that miR-203a negatively regulated WT1, BMI1, and XIAP expression (Figures 5A–H).

### Overexpression of miR-203a Inhibits the Expression of the Target Genes WT1, BMI1, and XIAP and Upregulated the Expression of EGR1

**FIGURE 6:**
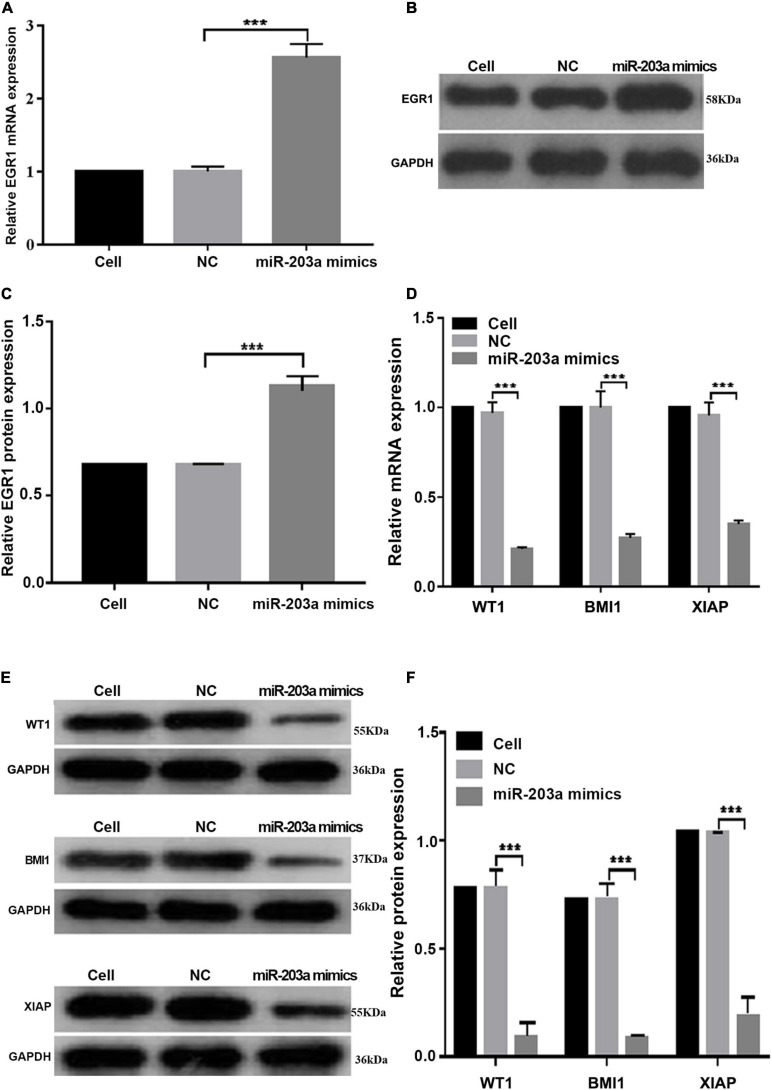
miR-203a overexpression in K562 cells increases the expression of EGR1, whereas the expression levels of the target genes WT1, BMI1, and XIAP are downregulated **(A)** After miR-203a was overexpressed in K562 cells, the relative expression of the EGR1 gene was detected by RT-PCR. ***P < 0.05 compared with the NC groups, *n* = 3. **(B)** After miR-203a was overexpressed in K562 cells, the relative expression of EGR1 protein was detected by western blotting. **(C)** Relative expression of EGR1 protein. ***P < 0.05 compared with the NC groups, *n* = 3. **(D)** After miR-203a was over expressed in K562 cells, RT-PCR was used to detect the relative expression of the WT1, BMI1, and XIAP genes. ***P < 0.05 compared with the NC groups, *n* = 3. **(E)** After miR-203a was overexpressed in K562 cells, the relative protein expression of WT1, BMI1, and XIAP was detected by western blotting. **(F)** Relative protein expression of WT1, BMI1, and XIAP, ***P < 0.05 compared with the NC groups, *n* = 3.

At 48 h after the overexpression of miR-203a in K562 cells, EGR1 expression was upregulated, whereas the gene and protein levels of the target genes WT1, BMI1, and XIAP were downregulated (Figure 6). These results showed that miR-203a positively regulated EGR1 expression, thus inhibiting the expression of the target genes WT1, BMI1, and XIAP.

### miR-203a Overexpression Inhibits K562 Cell Proliferation and Co-Transfection With pcDNA3.1 + WT1, pcDNA3.1 + BMI1, and pcDNA3.1 + XIAP Offsets the Anticancer Role of miR-203a

**FIGURE 7:**
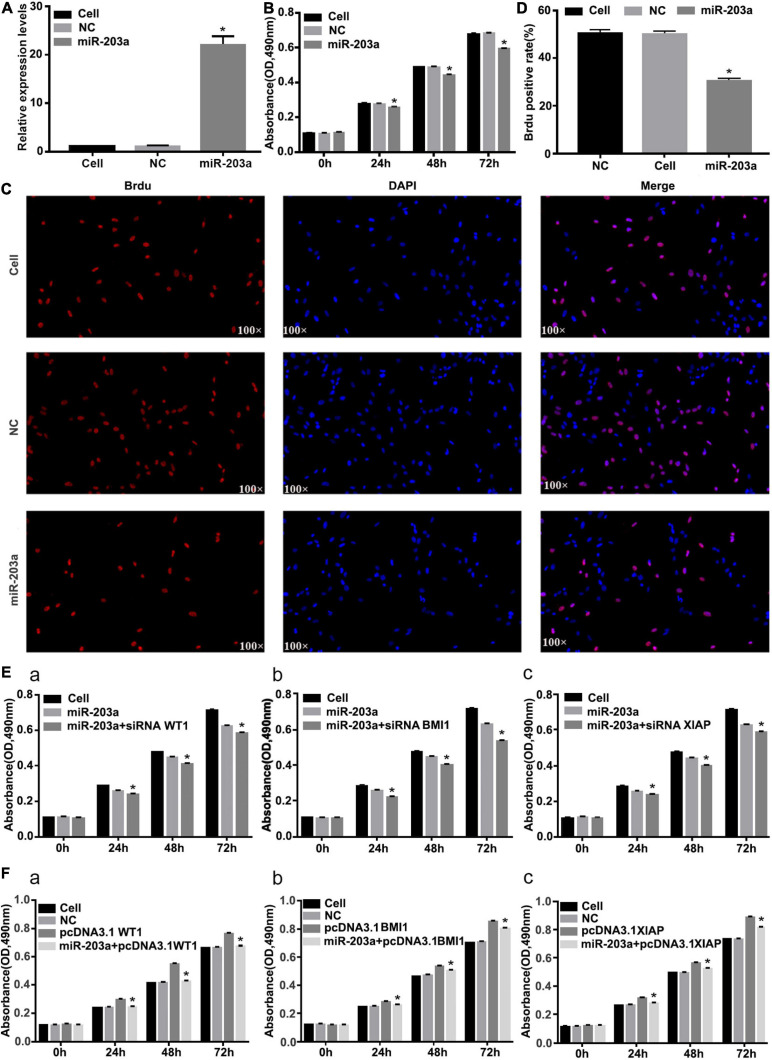
Overexpression of miR-203a inhibits the proliferation of K562 cells, whereas WT1, BMI1, and XIAP overexpression can counteract the antitumor effect of miR-203a **(A)** After miR-203a was overexpressed in K562 cells for 48 h, RT-PCR was used to detect the relative expression of miR-203a, *P < 0.05 compared with the NC group, *n* = 3. **(B)** After miR-203a was overexpressed in K562 cells for 24, 48, and 72 h, the cell proliferation rate was detected by an MTT assay. *P < 0.05 compared with the NC and cell groups, *n* = 3. **(C)** A BrdU assay was used to detect the proliferation of K562 cells. **(D)** BrdU-positive cell rate. **(E)** K562 cells overexpressing miR-203a were co-transfected with si-BMI1, *P < 0.05 compared with the miR-203a and cell groups, *n* = 3 (a), si-WT1 (b), or si-XIAP (c), and the cell proliferation rate was compared. **(F)** K562 cells overexpressing miR-203a were co-transfected with pcDNA3.1 + WT1, *P < 0.05 compared with the NC, cell groups and pcDNA3.1 group, *n* = 3. (a), pcDNA3.1 + BMI1 (b), or pcDNA3.1 + XIAP (c), and the cell proliferation rate was compared.

After miR-203a was overexpressed in K562 cells, the cell proliferation rate was significantly inhibited (Figures 7A–D). After co-transfection of si-WT1, si-BMI1, or si-XIAP with miR-203a mimic, the proliferation of K562 cells was significantly inhibited (Figure 7E). After co-transfection of pcDNA3.1 + WT1, pcDNA3.1 + BMI1, or pcDNA3.1 + XIAP with miR-203a mimic, the proliferation of K562 cells was promoted, thereby offsetting the anticancer effect of miR-203a (Figure 7F).

### EGR1 Positively Regulates miR-203a Expression, Thus Relieving the Inhibition of Translation by miR-203a on the Target Genes WT1, BMI1, and XIAP

**FIGURE 8:**
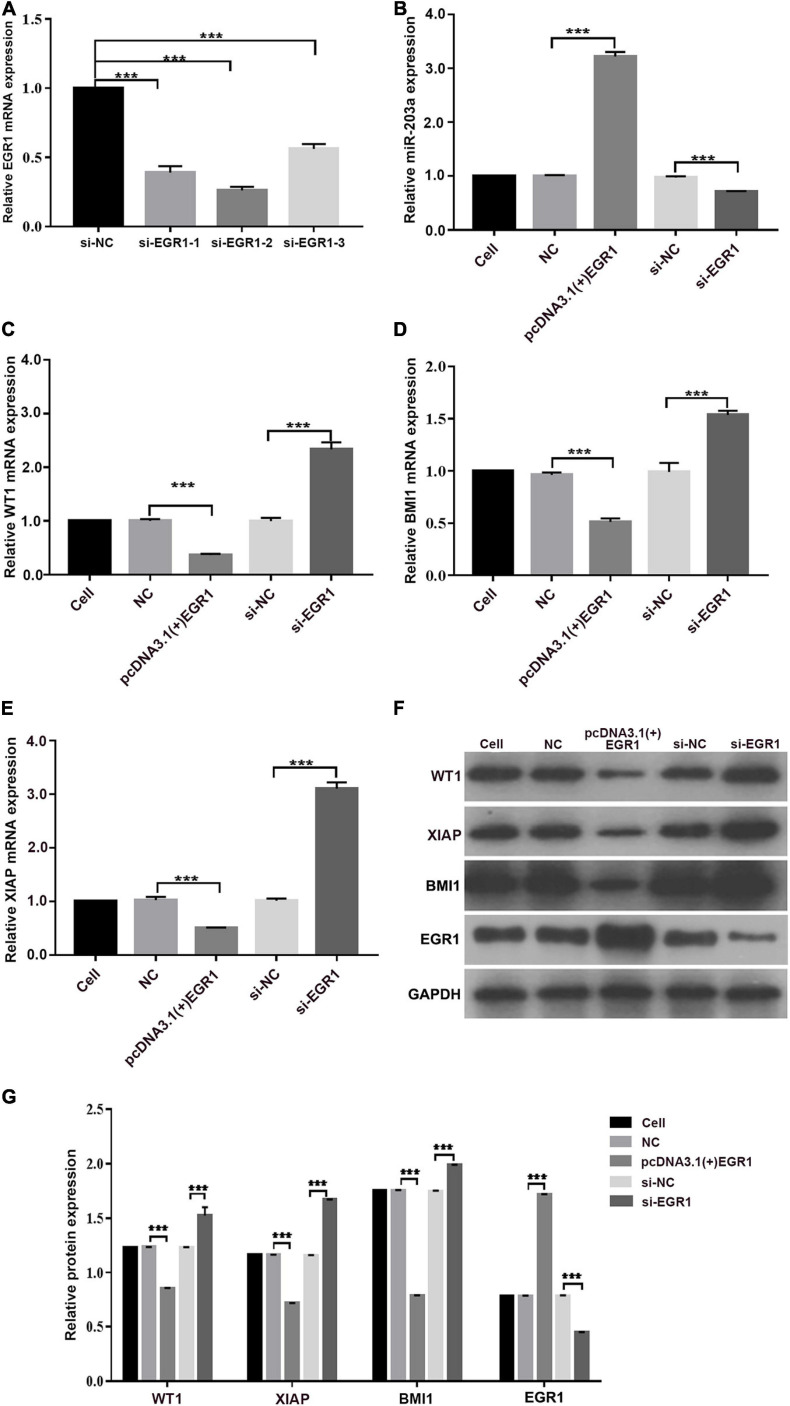
EGR1 positively regulates miR-203a expression, thus affecting the inhibition of translation by miR-203a on the target genes WT1, BMI1, and XIAP **(A)** Screening of three different siRNAs targeting EGR1. ***P < 0.05 compared with the si-NC group, *n* = 3. **(B)** After the overexpression or knockdown of EGR1 expression, the relative expression of miR-203a was assessed. ***P < 0.05 compared with the si-NC and NC groups, *n* = 3. **(C)** After the overexpression or knockdown of EGR1 expression, the relative expression of WT1 was detected by RT-PCR. ***P < 0.05 compared with the si-NC and NC groups, *n* = 3. **(D)** After the overexpression or knockdown of EGR1 expression, the relative expression of BMI-1 was detected by RT-PCR. ***P < 0.05 compared with the si-NC and NC groups, *n* = 3. **(E)** After the overexpression or knockdown of EGR1 expression, the relative expression of XIAP was detected by RT-PCR. ***P < 0.05 compared with the si-NC and NC groups, *n* = 3. **(F)** Relative protein expression was detected by western blotting. **(G)** Relative quantitative expression of WT1, BMI1, EGR1, and XIAP protein. ***P < 0.05 compared with the si-NC and NC groups, *n* = 3.

In K562 cells, miR-203a expression was decreased when EGR1 expression was knocked down, and miR-203a expression was increased when EGR1 was overexpressed, showing a positive regulatory relationship between EGR1 and miR-203a (Figures 8A,B). The expression of WT1, BMI1, and XIAP gene and protein was downregulated after overexpressing EGR1 in K562 cells; when EGR1 expression was decreased, WT1, BMI1, and XIAP gene and protein expression was upregulated (Figures 8C–G). These results indicated that the transcription factor EGR1 positively regulates miR-203a expression, thus affecting the inhibition of translation on the miR-203a target genes WT1, BMI1, and XIAP.

## Discussion

The miR-203a gene is located on chromosome 14q32.33, which is an unstable region of the chromosome that encodes approximately 12% of known miRNAs. The abnormal expression of miR-203a is related to the occurrence and development of gastric cancer, ovarian cancer, colon cancer, lung cancer, CML, and other tumors (Funamizu et al., 2015; Zhao et al., 2015; Liang et al., 2016; You et al., 2016). Methylation of miR-203 was observed in Ph negative leukemia patients. The methylation patterns of the miR-203 gene locus have been suggested as a potential biomarker for CML diagnosis (Chim et al., 2011). miR-203 affects the proliferation of K562 cells by targeting BCR-ABR expression (Bueno et al., 2008). miR-203a promotes the early apoptosis of K562 cells by activating caspase-3 and caspase-9, which are key enzymes of the mitochondrial apoptosis pathway, and it improves the sensitivity of K562 cells to chemotherapy drugs (He et al., 2013). However, the molecular mechanism by which miR-203a regulates the development of CML is not clear and needs further study.

In the present study, to examine the potential molecular mechanism of miR-203a expression in CML cells, we constructed double luciferase reporter gene vectors, performed rescue assays, and used biological information software prediction to confirm that miR-203a negatively regulated the expression of WT1, BMI1, and XIAP, which affected the proliferation of K562 cells.

The WT1 gene is highly expressed in human leukemia cells, and not only plays an important role in the proliferation and differentiation of leukemia cells but is also associated with the occurrence, development, and prognosis of leukemia. At present, the WT1 gene is used as the basis for the initial diagnosis of leukemia, the judgment of prognosis, and the need for hematopoietic stem cell transplantation (Ariyaratana and Loeb, 2007; Liu et al., 2014). The BMI1 gene plays an important role in regulating the proliferation of normal cells and leukemia stem cells. BMI1 expression is related to the degree of malignancy (Tong et al., 2011). XIAP, as the strongest anti-apoptotic factor in the inhibitors of apoptosis family, is expressed at a significantly higher level in patients with CML than in normal subjects (Zhao and Ma, 2013). Knockdown of XIAP effectively inhibits the proliferation of K562 cells, increases the apoptosis rate, and effectively reverses CML resistance (Lima et al., 2006; Seca et al., 2011).

miR-203a functions by interacting with a variety of mRNA targets, such as suppressing bladder cancer cell growth by targeting Twist1 (Shen et al., 2018), suppressing the proliferation and metastasis of hepatocellular carcinoma by targeting the oncogene ADAM9 and the oncogenic long non-coding RNA HULC (Iino et al., 2019), suppressing cell proliferation, migration, and invasion in colorectal cancer by targeting EIF5A2 (Wan et al., 2016), and increasing the sensitivity of K562 cells to S_2_O_3_ by downregulating the expression of BCR-ABL (He et al., 2013). Clarifying the expression levels of miR-203a in CML and the regulatory relationship between miR-203a and its target genes may provide a new intervention target for tumor gene therapy.

The regulation of gene expression at the pre-transcriptional level by miRNAs can involve transcription factors, in which their interaction with the target gene is adjusted, constituting a gene expression regulation network (Deng et al., 2016). In the miRNA-transcription factor common target gene regulation process, transcription factors can regulate miRNAs, and in turn can be regulated by miRNAs, forming a feed-forward loop (Yang et al., 2018). In the present study, the transcription factor EGR1 was found to bind to the promoter region of miR-203a, and we confirmed that the transcription start site of miR-203a was at the G nucleotide located at −339 bp in the upstream promoter region. EGR1 positively regulated miR-203a expression, thus relieving the inhibition of translation by miR-203a on WT1, BMI1, and XIAP. EGR1 targets and regulates multiple miRNAs in CML K562 cells, thereby affecting the effect of these miRNAs on the expression of downstream target genes and affecting cell proliferation and apoptosis. miRNAs and transcription factors exert a precise regulatory effect on the expression of target genes in intact transcriptional regulatory networks (Mullany et al., 2018). For example, GATA6 suppresses gastric cancer cell migration and metastasis via the miR-520b-mediated repression of CREB1. Therefore, targeting the GATA6/miR-520b/CREB1 axis may be an effective approach for the treatment of gastric cancer (Liu et al., 2019). CDX2 inhibits the expression of miR-145-5p, thereby relieving the inhibitory effect of miR-145-5p on the translation of SENP1 and affecting the invasion and migration of prostate cancer cells (He et al., 2019). Thus, transcription factors interact with miRNAs to form a feed-back loop to regulate gene expression.

## Conclusion

In conclusion, the present study revealed the relationship between the EGR1-miR-203a-WT1/BMI1/XIAP regulatory network and transcriptional and post-transcriptional regulation. The results showed that miR-203a expression was positively regulated by the transcription factor EGR1, which affected the expression of downstream target genes (WT1, BMI1, and XIAP). EGR1 could bind to the promoter region of miR-203a to relieve the inhibition of translation by miR-203a on the target genes WT1, BMI1, and XIAP and affect the proliferation of K562 cells. These findings have generated clues for revealing the molecular mechanism of CML and may provide a new experimental basis and methods for targeted gene therapy of leukemia.

## Data Availability Statement

The raw data supporting the conclusions of this article will be made available by the authors, without undue reservation.

## Ethics Statement

The studies involving human participants were reviewed and approved by the Ethics Committee of Panyu District Central Hospital. The patients/participants provided their written informed consent to participate in this study.

## Author Contributions

JH, ZH, ZA, and YL performed the experiments. JZ analyzed the data. JH and ZH wrote the manuscript. SH and YL designed the study. MH, GL, and HQ revised the manuscript. XX and YL provided the reagents. All authors read and approved the final manuscript.

## Conflict of Interest

The authors declare that the research was conducted in the absence of any commercial or financial relationships that could be construed as a potential conflict of interest.

## Footnotes

1 http://rna.sysu.edu.cn/chipbase/

2 https://www.ncbi.nlm.nih.gov/pubmed/

3 https://www.ncbi.nlm.nih.gov/nuccore/NR_029620.1

## Abbreviations

BMI1: B cell-specific Moloney murine leukemia virus integration site 1
ChIP: chromatin immunoprecipitation
CML: chronic myeloid leukemia
EGR1: early growth response 1
miRNA (miR): microRNA
NC: negative control
PBS: phosphate-buffered saline
RACE: rapid amplification of cDNA ends
RT-PCR: real-time polymerase chain reaction
siRNA: small interfering RNA
UTR: untranslated region
WT1: Wilms tumor 1
XIAP: X-linked inhibitor of apoptosis protein.
